# The Role of Thromboxane in the Course and Treatment of Ischemic Stroke: Review

**DOI:** 10.3390/ijms222111644

**Published:** 2021-10-28

**Authors:** Małgorzata Szczuko, Igor Kozioł, Dariusz Kotlęga, Jacek Brodowski, Arleta Drozd

**Affiliations:** 1 Department of Human Nutrition and Metabolomics, Pomeranian Medical University, 72-460 Szczecin, Poland; igorkoziol11@gmail.com (I.K.); arleta.drozd@pum.edu.pl (A.D.); 2 Department of Pharmacology and Toxicology, University of Zielona Góra, 65-417 Zielona Góra, Poland; dkotlega@uz.zgora.pl; 3 Primary Care Department, Pomeranian Medical University, Żołnierska 48, 71-210 Szczecin, Poland; Jacek.brodowski@pum.edu.pl

**Keywords:** thromboxane, cerebral stroke, acetylsalicylic acid

## Abstract

Cardiovascular diseases are currently among the leading causes of morbidity and mortality in many developed countries. They are distinguished by chronic and latent development, a course with stages of worsening of symptoms and a period of improvement, and a constant potential threat to life. One of the most important disorders in cardiovascular disease is ischemic stroke. The causes of ischemic stroke can be divided into non-modifiable and modifiable causes. One treatment modality from a neurological point of view is acetylsalicylic acid (ASA), which blocks cyclooxygenase and, thus, thromboxane synthesis. The legitimacy of its administration does not raise any doubts in the case of the acute phase of stroke in patients in whom thrombolytic treatment cannot be initiated. The measurement of thromboxane B2 (TxB2) in serum (a stable metabolic product of TxA2) is the only test that measures the effect of aspirin on the activity of COX-1 in platelets. Measurement of thromboxane B2 may be a potential biomarker of vascular disease risk in patients treated with aspirin. The aim of this study is to present the role of thromboxane B2 in ischemic stroke and to present effective therapies for the treatment of ischemic stroke. Scientific articles from the PubMed database were used for the work, which were selected on the basis of a search for “thromboxane and stroke”. Subsequently, a restriction was introduced for works older than 10 years, those concerning animals, and those without full text access. Ultimately, 58 articles were selected. It was shown that a high concentration of TXB2 may be a risk factor for ischemic stroke or ischemic heart disease. However, there is insufficient evidence to suggest that thromboxane could be used in clinical practice as a marker of ischemic stroke. The inclusion of ASA in the prevention of stroke has a beneficial effect that is associated with the effect on thromboxane. However, its insufficient power in 25% or even 50% of the population should be taken into account. An alternative and/or additional therapy could be a selective antagonist of the thromboxane receptor. Thromboxane A2 production is inhibited by estrogen; therefore, the risk of CVD after the menopause and among men is higher. More research is needed in this area.

## 1. Introduction

Stroke is one of the most common causes of death and disability in the world. In 2013, the American Heart Association/American Stroke Association updated the definition of stroke to include silent infarcts (including cerebral, spinal, and retinal). The rationale for this change was the transition to a radiological demonstration (tissue definition) of an infarction [1]. Of the many types of stroke, 80% of cases are ischemic strokes [2]. Its main cause is cardiovascular diseases (CVD), in which increased biosynthesis of TxA2 and isoprostanes, as well as increased activation of the thromboxane A2 receptor (TXA2R), are observed [3]. Thromboxane A2 (TXA2) is the predominant product of human platelet cyclooxygenase. It shows a strong pro-aggregation effect and is the main factor constricting blood vessels. This compound plays a key role in the pathophysiological mechanisms of stroke, promoting inflammation, apoptosis, excitotoxicity, and peri-infarct depolarization [4].

The literature has indicated the significant role of thromboxane in myocardial infarction, in which it induces platelet aggregation and narrows blood vessels [5,6,7]. Thromboxane, along with isoprostanes and PGI2, also promotes the initiation and progression of atherosclerosis by controlling platelet activation and the interaction of leukocytes with endothelial cells [8]. A correlation has also been demonstrated between the increase in circulating plasma TXA2 and the hypersensitivity of the coronary arteries to ergonovine maleate in patients with angina. This suggests the possible role of increased TXA2 production in the exacerbation of coronary spasticity [9].

Currently, there are also increasing reports of acute cerebrovascular disease (CVD) resulting from SARS-CoV-2 virus infection, including both acute ischemic stroke (AIS) and intracerebral hemorrhage (ICH) [10]. CVD is also a factor that increases the mortality and complications of COVID-19 [10]. Researchers have suggested that thromboxane, along with other eicosanoids, is largely responsible for the activation of the “cytokine storm” mediated by IL-6, IL-1, and TNF alpha. [11]. Thrombotic complications in COVID-19 patients are common and contribute to organ failure and death. The etiology of stroke is cryptogenic and twice as high in COVID-19 patients as that of general population. SARS-CoV-2 is potentially a higher precipitating factor for acute ischemic stroke than other classic respiratory infections such as influenza, possibly via immune-mediated coagulopathy [12]. The interaction between thromboxane and COVID-19 stroke has not been studied so far [12].

The aim of the study is to present the current knowledge on the pathomechanisms of ischemic stroke, with particular emphasis on the role of thromboxane B2. We also analyzed data from the literature on the possibility of using COX-1 and/or COX-2 inhibitors, with particular emphasis on acetylsalicylic acid (ASA) as an inflammatory mediator, both in the case of cerebral infarction and as a drug that reduces thromboxane levels. On the other hand, exploring whether thromboxane B2 could be a marker of ischemic stroke and the characterization of other possible blockers of thromboxane synthesis were additional objectives of the study.

## 2. Thromboxane and Stroke

The action of thromboxane B2 is not limited to the circulatory system [13]. Urinary 11-dehydro-thromboxane has been described as a potential predictive biomarker of major adverse cardiovascular events in patients with a high cardiac risk and provides prognostic information on the performance of the left ventricle [14]. TXA2 is involved in nephritis and kidney nephrotic syndrome by stimulating mesangial cells, causing them to contract, promoting proliferation, changing ion flow, and increasing the synthesis of fibronectin, laminin, tissue collagen plasminogen activator (tPA), plasminogen activator inhibitor-1 (PAI-1), and initiation of the synthesis of growth factors [15]. Liver damage after hepatic stress (e.g., endotoxemia, non-reperfusion liver ischemia, hepatectomy, liver transplantation, and also NFLD) is associated with increased synthesis of thromboxane B2 in the liver, which induces vasoconstriction, platelet aggregation, and induction of leukocyte adhesion and promotes oxidative stress [16]. TXA2-related oxidative stress causes stimulation of angiogenesis, endothelial cell migration, and angiotensin II-induced neovascularization and is observed during the neoplastic process. It is also believed that TXA2 promotes the metastasis of neoplastic cells, including in ovarian and colon cancer [17]. In the context of diseases and blood vessels the effect is on the blockage of the blood vessels and the blockage of the arteries [17].

Due to the broad mechanisms of action of the arachidonic acid pathway, blocking the cyclooxygenase pathway with COX-1 and COX-2 inhibitors is a commonly used treatment method [5]. However, due to the occurrence of unfavorable side effects, such as gastrointestinal bleeding, researchers’ attention has been focused on targeted blocking of specific pathways in the synthesis of AA metabolites, particularly thromboxane A2 [7,8].

### 2.1. Thromboxane Synthesis and Its Mechanisms of Action

Arachidonic acid (AA, 20: 4n-6) is the major substrate for the synthesis of eicosanoids, including thromboxane A2 (TXA2) [6]. The highest concentrations of arachidonic acid are found in the brain, muscles, liver, spleen, and retina. Circulating free AA concentrations are usually very low due to binding by albumin. Endogenous AA production occurs mainly through the release of AA from cell membrane phospholipids [18]. This process is catalyzed by phospholipase A2 (PLA2) related enzymes and is induced by a variety of cell-activating signals, including stimulation of inflammation or infection of the tumor necrosis factor receptor (TNFR) and stimulation of Toll-like receptor 4 (TLR4) [19]. Proper metabolism of arachidonic acid is essential for organism homeostasis. AA itself gives flexibility and fluidity to cell membranes, and serves as a lipid messenger in cell signaling. AA changes the flow in ion channels and controls the voltage of the proton pump, as well as modulating sodium channels in the heart, which are the main factors affecting its excitability. Modulation of the proton pumps and pH is also necessary for phagocytes to produce reactive oxygen species (ROS) via NADPH oxidase; therefore, free AA induces oxidative stress. Oxidative stress itself has dramatic consequences for AA metabolism, as it alters PLA2 release from AA, promotes AA self-oxidation, and induces COX-2 overexpression. The activation of ROS by free AA is a major factor in the regulation of apoptosis and possible neoplastic activity, which is of particular interest in cardiovascular and metabolic diseases, since oxidative stress is an important factor in the pathogenesis of DM, hypertension, fatty liver, atherosclerosis, and heart disease [20].

There are five known pathways for converting free arachidonic acid into specific derivatives. In the first stage, common to all derivatives, cyclooxygenase 1 (COX-1) and 2 (COX-2) (prostaglandin G/H synthase) catalyze the formation of PGH2, which is then converted by the appropriate synthases to thromboxane A2 (TXA2), prostacyclin (PGI2), and several prostaglandins (PGD2, PGF2, and PGE2) [8].

COX-1 is expressed and is found in all tissues that are susceptible to lipopolysaccharide (LPS)-induced inflammation. TXA2 and prostaglandins (PG) such as prostaglandin D2 (PGD2) alter vascular tone and mediate platelet aggregation. COX-2 is found in macrophages and endothelial cells, and is highly expressed in the brain and kidneys, and is stimulated by bacterial endotoxins, growth factors, hormones, and several cytokines. Its major metabolites, prostaglandin E2 (PGE2), PGI2, PGD2, and prostaglandin F2 alpha (PGF2α), regulate the host immune response, vascular tone regulation, thrombus formation, pain perception, and female fertility and are involved in neurodegeneration. Moreover, COX facilitates the synthesis of prostaglandins in the human brain and heart [21].

**Figure 1:**
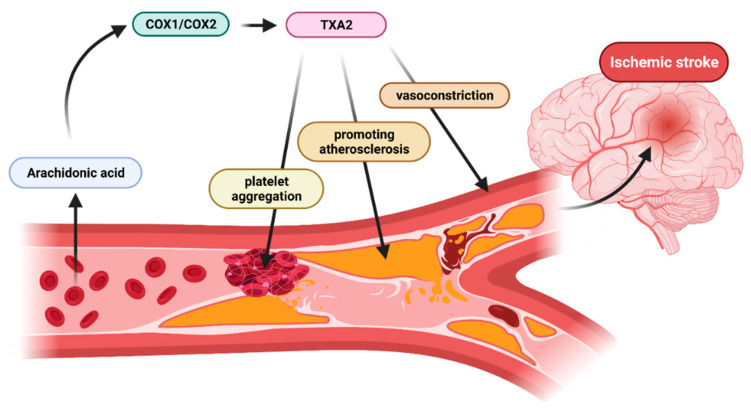
Schematic representation of the mechanism of action of thromboxane in patients with atherosclerosis. Thromboxane induces platelet aggregation leading to clot formation, constriction of blood vessels and plaque deposition (created with BioRender.com https://app.biorender.com/, accessed on 23 October 2021).

TXA2 controls biological processes owing to the presence of the TP receptor on the cell surface. The TXA2 receptor is activated by TAX2 and isoprostanes. Increased levels of TXA2 and its receptor are observed in many cardiovascular and inflammatory diseases. Enhanced TX generation may be explained by the mechanisms being relatively insensitive to aspirin. Therefore, thromboxane synthase inhibitors (TXSI) and thromboxane receptor antagonists (TXRA) have the potential to prove more effective than aspirin due to their different mechanisms of action along the pathway of TXA2 in preventing thrombotic events in cardiovascular disease [22]. The diagram of TXA2 activity leading to stroke in people with CVD is shown in the Figure 1.

### 2.2. Natural Regulatory Substances Preventing Thromboxane Synthesis

Research was also conducted on the effect of natural substances that reduce thromboxane synthesis. Correlations were demonstrated between several of them.

Catechol suppressed COX-1 and COX-2 activity by approximately 30–50%. It also inhibited IL-1β-induced PGE2 production, but not COX-2 expression of fibroblasts. These results suggested that catechol exhibited antiplatelet and anti-inflammatory effects, which were mediated by the inhibition of COX, ROS, and TXA2 production, as well as ERK/p38 phosphorylation [23].

Hydroxy-cinnamic acids (cinnamic acid derivatives), especially caffeic, ferulic, and chlorogenic acids, present various biological activities, including antioxidant, anti-inflammatory, anticancer, antiatherogenic, and antimicrobial activities. Additionally, the cinnamoyl moiety is present in various drugs such as ozagrel, cinanserin, and tranilast, where it acts as an inhibitor of thromboxane A2 synthase [24].

There is evidence from interventional trials that consumption of dietary polyphenols may improve cardiovascular health by virtue of their antiplatelet properties. Several researchers are also pointing to flavonoids as antiplatelet agents [25].

Other substances are also known that may play a role in blocking the synthesis of thromboxane but have not been studied in the context of ischemic stroke. One of them is substance P (SP), belonging to the tachykinin neuropeptide family. Substance P is a potent vasodilator dependent on nitric oxide release [26]. SP initiates the expression of cytokines and stimulates phagocytic and chemotactic capacity, as well as increased PGE2, and thromboxane B2 synthesis [27]. A mixture of very high molecular weight aliphatic acids (D-003) was purified from sugar cane wax. Inhibition of platelet aggregation induced by D-003 is associated with a reduction of thromboxane B2 (TXB2) and an increase of prostacyclin (Pgl2) serum levels in healthy volunteers [28]. There are no studies comparing the effectiveness of natural substances against ASA and the thromboxane receptor. It seems that natural substances with antiplatelet properties should be treated as prevention and not treatment against stroke.

### 2.3. Thromboxane, Inflammation, and Ischemic Stroke

Thrombosis of the arteries resulting from atherosclerosis is the leading cause of stroke. Platelet aggregation plays a key role in the development of atherosclerosis and thrombosis, with TXA2 stimulating this process. In the physiological state, thromboxane A2 (TXA2), together with prostacyclin (PGI2), maintain vascular homeostasis and platelet aggregation. PGI2 is a vasodilator and platelet aggregation inhibitor, and TXA2 is a vasoconstriction and platelet aggregation promoter. Due to their opposing functions, PGI2 and TxA2 must be carefully balanced. An imbalance in PGI2 or TXA2 production is associated with the pathophysiology of myocardial infarction, atherosclerosis, and CI stroke [29].

The expression of COX-1 and COX-2 in the arachidonic acid pathway is increased in synovial inflammation, as well as in the endothelium, vascular smooth muscle cells, and macrophages in atherosclerotic lesions in humans. TxA2 is the most abundant product of COX-1 arachidonic acid metabolism in mature human platelets, while monocytes/macrophages synthesize TXA2 using the COX-2 enzyme [30]. The synthesis of thromboxane A2 (TXA2) is further catalyzed by thromboxane synthase A1 (TBXAS1) [30].

Identifying the genetic factors of a stroke can play an important role in the early diagnosis of vulnerability to stroke in high-risk individuals before symptoms are detected, in order to allow appropriate intervention. The gene encoding TBXAS1 was tested as a candidate gene involved in atherosclerotic plaque formation. The *TT SNP TBXAS1 rs2267682* genotype was selected in the Chinese population as one that increased susceptibility to ischemic stroke. The TT genotype and *rs2267682 T* allele probably enhance promoter activity and lead to upregulation of TBXAS1 expression [31]. Moreover, genetic variants of PTGS2, TXA2R, and TXAS1 are associated with carotid plaque vulnerability, platelet activation, and TXA2 levels in ischemic stroke patients [32].

During an ischemic stroke, nerve tissue is damaged by disturbed homeostasis of calcium ions, inhibited energy production, excitotoxicity, oxidative stress, and the emerging and progressing inflammatory process; thromboxane A2 is involved in all these processes [33]. The cascade of events is as follows: the lack of oxygen supply to the neurons leads to blockage of some ATP channels. Inhibition of ATP production impairs the operation of the ATPase-dependent sodium–potassium pump, thus blocking the removal of sodium ions from the cell. The energy deficit contributes to the disturbance of the depolarization of the cell membrane of the neuron, which results in an increase in the concentration of sodium, calcium, and chloride ions in the cell and potassium ions in the intercellular space [33].

At the same time, calcium ions are released from intracellular stores, particularly from the axoplasmic reticulum. The increased concentration of calcium ions is a significant cause of ischemic stroke because it stimulates calcium-dependent enzymes such as protein kinase C, phospholipase A2, and protein kinase, as well as many other protease and endonuclease processes causing apoptosis and necrosis [4]. In addition, nitrogen synthetase induced by the intensification of calcium ions, in combination with the abovementioned enzymes, can lead to damage and death of the nerve cells. Deprivation of myelin nerve fibers causes the discharge of potassium ions from the inside of the cell to the external environment as a result of the discovery of potassium channels. Nerve conduction in a demyelinated axon is a process that requires much more energy than the same process in a normal neuron [34].

Demyelination causes damage to the white matter of the brain, characterized by loss of the myelin sheath and death of the oligodendrocyte cells. This process contributes significantly to long-term sensorimotor and cognitive deficits because the adult brain has only a limited ability to regenerate oligodendrocytes and remyelinate axons. Inflammatory cytokines such as interleukin (IL)-1β, IL-2, interferon-γ (IFN-γ), and tumor necrosis factor-α (TNF-α) are key mediators of pathological changes in demyelinating disorders and participate in hemodynamic processes [35].

IL-1 also causes the thrombogenicity of platelets in non-endothelial cells by stimulating the formation of thromboxane A2, which is released into the inflamed environment. IL-1 is the most important immune molecule in causing fever because it is involved in the metabolism of arachidonic acid, which is produced by the vascular endothelial organs of the hypothalamus. The pathogenesis of thrombosis, vasculitis, and angiogenesis also includes mediating activation of the prostanoid thromboxane A2 receptor. IL-1 induction occurs in a variety of pathologies, which are also caused by pathogenic viral infections, including SARS-CoV-2, which provokes COVID-19. Activation of the macrophages by COVID-19 leads to the release of pro-inflammatory cytokines, metalloproteinases, and other proteolytic enzymes that can cause thrombus formation and severe respiratory dysfunction, along with a high risk of thrombotic complications and venous thromboembolism [36,37]. The SARS-CoV-2 virus is a very strong activator of inflammatory processes and intensifies the existing ones, which may explain the worse prognosis in patients with comorbidities [36,37].

Metalloproteinases are responsible for damage to the blood-brain barrier (BBB), thus increasing its permeability to leukocytes, which activate pro-inflammatory processes. Consequently, it causes swelling of the cerebral vessels, swelling of the nervous tissue, and an increase in intracranial pressure. The BBB acts as a barrier that separates the central nervous system (CNS) from peripheral tissues. Its permeability depends on the action of endothelial cells, whose cell membranes contain asymmetrically arranged transport proteins that facilitate the penetration of substances into the brain or their removal into the vessel lumen [38]. The increase in thromboxane levels also induces leukocyte aggregation and systemic inflammation, which is responsible for dramatic thrombus formation and organ dysfunction [5].

Understanding the mechanisms of the inflammatory process that accompanies stroke and finding a common denominator would bring real benefits for treating stroke, regardless of its etiology.

### 2.4. Changes in Thromboxane Levels In Vitro and In Vivo 

In 2019, Patrono and Rocca reviewed clinical trials over the past four decades, focusing on in vivo and ex vivo measurements of TXA2 metabolites as indicators of platelet activation and COX-1 activity. They analyzed the metabolic fate of TXB2 entering the main circulation by measuring 2,3-dinor-TXB2 excretion during exogenous TXB2 infusion in four healthy subjects who had received aspirin and randomly received a 6-hour infusion of the vehicle alone of TXB2 at 0.1, 1.0, and 5.0 ng/kg/min. Plasma TXB2 and urine 2,3-dinor-TXB2 were measured before, during, and up to 24 h post-infusion and during the aspirin-free periods. Treatment with aspirin reduced baseline 2,3-dinor-TXB2 levels in urine by 80%. The fractional excretion of 2,3-dinor-TXB2 was independent of the TXB2 infusion rate over the 50-fold dose range and averaged 5.3% ± 0.8%. Introduction of the 2,3-dinor-TXB2 excretion rate measured during aspirin-free periods to a linear relationship between TXB2 infusion doses and metabolite excretion amounts above the control values allowed the estimation of the endogenous TXB2 entry rate to be 0.11 ng/kg/min. After the TXB2 infusion was stopped, the rate of disappearance from the systemic circulation was linear for the initial 10 min, with an apparent half-life of 7 min. This finding demonstrated the local nature of the synthesis and action of TXA2 on prostacyclin (PGI2). As with endothelial PGI2 synthesis, the maximal TXA2 biosynthetic capacity of human platelets far exceeds actual in vivo production. Thus, platelets in 1 mL of whole blood clotted for 1 h in vitro can simultaneously synthesize and release a similar amount of TXB2 to what is secreted into the systemic circulation in vivo [39].

Measuring the level of thromboxane synthesis in patients taking aspirin may be important for identifying people with resistance to aspirin. This is important because more than 15–25% of patients with arterial thrombosis may develop recurrent vascular events during treatment with ASA [40].

Due to safety concerns in humans, it is not possible to conduct a human TXA2 metabolic fate study and it has been an open question whether the enzymatic transformation of TXB2 to its major urinary metabolites accurately mirrors the in vivo metabolism of TXA2. For this reason, in 1989, Patrigiani and co-workers compared the metabolism of monkeys with exogenously administered TXA2 and TXB2. The primary conclusion from this study was that TXA2 and TXB2 are metabolized to 2,3-dinoro-TXB2 and 11-dehydro-TXB2 with comparable fractional conversion factors, suggesting that TXA2 is non-enzymatically hydrolyzed to TXB2 against enzymatic degradation by beta-oxidation and the 11-OH dehydrogenase pathways, and that the resulting urinary metabolites are a quantitative indicator of TXA2 biosynthesis in vivo. The researchers suggested that measuring the level of TXA derivatives may be a valuable non-invasive marker of TXA metabolism and for assessing the effectiveness of specific drugs [41].

### 2.5. Changes in Thromboxane Levels in CVD

A transient increase in 2,3-dinor-TXB2 and 11-dehydro-TXB2 excretion concentrations was reported in patients with acute coronary syndrome and was interpreted as reflecting repeated episodes of platelet activation. Transient changes in TXA2 biosynthesis, which were detected in patients with unstable angina, were accompanied by a concomitant increase in PGI2 biosynthesis, as reflected in the urinary excretion of 2,3-dinor-6-keto-PGF1α, indicating antiregulatory endothelial activation in this environment. However, patients with chronic stable angina did not show increased TXA2 biosynthesis, either at rest or after exercise-induced myocardial ischemia. An episodic increase in the excretion of the metabolite TXB2 was also observed in acute ischemic stroke patients, although with a lower frequency and duration than in acute coronary syndromes, perhaps reflecting the heterogeneity of the mechanisms responsible for ischemic stroke. Low doses of aspirin (50 mg daily) significantly blocked 11-dehydro-TXB2 excretion in this environment, reflecting the dominant source of TXA2 biosynthesis in platelets. Increased platelet activation was independently associated with the severity of stroke at admission. Rare episodes of platelet activation in patients with transient ischemic attack suggested that enhanced TXA2 biosynthesis was not secondary to cerebral ischemia [42].

Urinary prostanoid metabolites, such as 11-dehydro-TXB2, do not reflect a specific site of prostanoid biosynthesis. To characterize potential platelets compared with non-protein sources of TXA2 biosynthesis, researchers used the unique property of aspirin to produce selective, cumulative acetylation of platelet COX-1 when administered in small doses once daily. Renal COX isozymes, which were involved in the increased production of TXA2 in patients with systemic lupus erythematosus, were not inhibited by low-dose aspirin to any detectable degree. Significant reductions in urinary 11-dehydro-TXB2 excretion have been reported in patients with Type 2 diabetes after receiving 50 mg of aspirin each day for 1 week, with platelet turnover-dependent recovery to pre-treatment excretion within the next 10 days after aspirin discontinuation. This was consistent with the dominant role of platelets as the source of TXA2 biosynthesis in this environment [39]. Diabetes is one of the high risk factors for CVD, including stroke and atherosclerosis. Hyperglycemia activates the thromboxane A2 receptor to impair the integrity and function of BBB via the ROCK-PTEN-Akt-eNOS pathway [43].

In 2002, Eikelboom et al. investigated the effect of urinary 11-dehydro-TXB2 excretion as a potential biomarker of serious vascular disease risk in aspirin-treated high-risk patients enrolled in the HOPE (Heart Outcomes Prevention Evaluation) trial. After optimizing the baseline differences, the odds of developing myocardial infarction, stroke, or cardiovascular death increased with each increasing baseline quartile of 11-dehydro-TXB2, and patients in the upper quartile had levels approximately twofold higher than those in the lower quartile. A similar study by CHARISMA (Clopidogrel for High Atherothrombotic Risk and Ischemic Stabilization, Management, and Avoidance) largely confirmed the discovery that resulted from the previously described study. However, a major limitation of both the CHARISMA and HOPE studies was the lack of a control group for non-aspirin-treated subjects [44].

In 1982, Patrignani and colleagues determined the effect of aspirin on the activity of COX-1 in platelets by measuring the concentration of TXB2 in serum and the metabolites of TXB2 in urine. The maximum ability of platelet biosynthesis to generate TXA2 in response to endogenously formed thrombin during whole-blood clotting is reflected by TXB2 serum, while its measurement is widely used to assess the human pharmacology of platelet COX-1 inhibition in disease and health. Serum TXB2 measurements were also helpful in characterizing the pre-systemic nature of platelet COX-1 acetylation by aspirin, an important feature of aspirin’s pharmacodynamics contributing to its biochemical selectivity. It is essential that the TXB2 serum test is performed under conditions of immediate whole-blood incubation at 37 °C, which is a prerequisite for optimal thrombin production, arachidonic acid release, and its sequential conversion by platelet COX-1 and TX synthase with PGH2 and TXA2 production. By stimulating platelets induced with thrombin, the concentration of TXB2 in the blood increases within 60 min from 1 to 2 pg/mL (actual levels in the circulating plasma in vivo) to 300 to 400 ng/mL [39].

Excessive body weight is associated with biochemical evidence of persistently increased platelet activation and a high risk of atherothrombotic diseases. In 2018, Rothwell et al. analyzed the individual data of 117,279 patients who were recruited for 10 primary preventive examinations and noted that only in people weighing less than 70 kg were low doses of aspirin (75–100 mg) effective in inhibiting serious vascular events, but this dosage was not effective in nearly 50% of women and the vast majority of men weighing 70 kg or more. The beneficial effect of low dose aspirin was diminishing with increasing weight. However, higher doses (≥325 mg) of aspirin were only beneficial in people weighing 70 kg or more. On the other hand, even a low dose of aspirin received by individuals weighing less than 50 kg may increase the risk of death [45]. Although some work on aspirin did not confirm the change in efficacy based on body weight, the above data seem to confirm that the antiplatelet effect of aspirin (as reflected in serum TXB2 measurement) is correlated with body size [46]. Parameters such as an increase in body size and too much fat and the resulting changes in liver function may contribute to a decrease in the bioavailability of the lipophilic drug aspirin. In a 2019 study by Petrucci measuring serum TXB2 at the end of the 24-hour interval between aspirin doses in 100 patients with a wide body mass index range, an important association was noted between body size, expressed as BMI, and serum TXB2 values. Therefore, the classical regimen of 100 mg aspirin once daily is likely to be insufficient to fully maintain platelet COX-1 activity in obese patients within the 24-hour dosing interval [41]. It is worth noting that beneficial effects of higher doses of aspirin in overweight patients can be related to the age and gender. Moreover, potential aspirin side-effects should be taken into consideration, especially in regard to gastrointestinal bleedings, that also seem to be associated with the weight [47].

Another issue that should be mentioned according to the weight and aspirin use, is the fact that aspirin responsiveness measured via the generation of TXB2 could be associated with the type of aspirin formulations. An adequate inhibition of serum TXB2 was observed in individuals <90 kg treated with the enteric-coated 75 mg dose aspirin, while patients weighted >90 kg responded better to plain aspirin. Regardless of the type of aspirin taken, in patients with higher body weight, increasing the dose of aspirin to 150 mg daily resulted in greater effectiveness of the action on thromboxane [48].

If we look at four decades of research into the biosynthesis and inhibition of thromboxane, the translational aspects of this research stand out in at least three areas. By analyzing thromboxane biosynthesis and inhibition studies over the past four decades, it can be seen that the measurement of urinary TX excretion, which is a non-invasive marker of in vivo platelet stimulation, plays an important role in determining the clinical conditions for evaluating the safety and efficacy of antiplatelet therapy. Examples concerning acute coronary syndrome and ischemic stroke are of particular importance. The specific low cardio-protective dose of aspirin under these conditions presumably reflects the important pathophysiological role of the persistently or transiently increased activation of platelets, which is explained by the TX measurement.

### 2.6. Application of Acetylsalicylic Acid

Acetylsalicylic acid (ASA) is a drug that has been used in humans for many years. Initially, it was used in the treatment of pain and it was also used as an antipyretic. Its widespread use in cardiological prevention came as a result of the publication of the first randomized controlled trial in 1974 [48]. The mechanism of action of acetylsalicylic acid is based primarily on the inhibition of two isoenzymes: cyclooxygenase 1 and cyclooxygenase 2. COX-1 has a protective effect on the gastric mucosa and protects blood vessels. COX-2 is involved in the inflammatory response and the formation of prostaglandins, thromboxane, and prostacyclin. Prostacyclins, along with leukotriene and thromboxane, are involved in platelet aggregation, fibrinolysis, and the process of contraction and relaxation of blood vessels, as well as the inflammatory response. The influence of ASA on all types of COX is irreversible. By affecting platelets, COX production is inhibited, resulting in a thrombocyte that does not synthesize COX until the end of the cell’s life (approximately 10 days) without this enzyme activity [49].

ASA is absorbed in the stomach and in the upper part of the small intestine, but it can also occur in the mouth when the tablet is chewed, rather than swallowed, which helps to produce levels in the blood that inhibit platelet synthesis in a shorter time. In the case of coated tablets, the highest concentrations of ASA are achieved after about 3–4 h after ingestion of the drug, while uncoated tablets allow this to be achieved after about 30–40 min. ASA is completely excreted by the kidneys; therefore, urine pH plays an important role in its elimination. The decreased excretion of salicylates is caused by acidification of the urine, while its alkalization increases the renal clearance of ASA [50].

Aspirin has a wide range of uses in the prevention of patients with known cardiovascular disease. The preventive effect is mainly related to its ability to inactivate COX-1, which leads to the inhibition of the synthesis of thromboxane 2 (TXA2). In addition, platelets are unable to produce the new COX enzyme due to a lack of cell nuclei. Consequently, inhibition of COX-1 is sustained over the lifetime of platelets, i.e., 10 days. Patients are instructed to take aspirin upon awakening at doses of 75–100 mg once daily. However, 15% of these patients experience a stroke or cardiovascular event while taking aspirin [51]. The possible cause of cardiovascular disorders despite the use of aspirin may be related to the pharmacokinetic characteristics of aspirin. The production of new platelets (reticular plaques) may explain this process. The production and release of these reticular platelets by megakaryocytes follows a circadian rhythm, with the maximum release occurring late at night and early in the morning. In addition, previous studies have shown that most ischemic events occur at these times. Therefore, these hours may be necessary for adequate platelet inhibition. The study by Racce et al. concluded that the once-a-day aspirin regimen had the lowest level of platelet inhibition compared with the once-a-day evening regimen and the twice-daily regimen with the dose reduced by half [52].

Patients with systemic lupus erythematosus (SLE) are at a greater risk of thrombotic complications than the general population. The mechanisms underlying this increased risk are unclear, but include both the severity of atherosclerosis and the propensity toward thrombosis. As a result, in addition to intervening to reduce atherosclerosis, many SLE patients also receive aspirin to stop thrombosis. Low doses of aspirin inhibit the production of TxA2 platelets almost completely in healthy people, and a dose of 81–100 mg/day is widely recommended for the prevention of myocardial infarction [53]. However, not all patients respond adequately to aspirin, and an inefficient effect of aspirin has been observed in essential thrombocythemia, in coronary artery disease, and in metabolic syndrome, among others [53].

The measurement of serum thromboxane B2, a stable metabolic product of TxA2, is the only test that measures the effect of aspirin on platelet COX-1 activity. Measurement of serum thromboxane B2 (sTXB2) TxB2 in whole blood that has been allowed to clot can determine the maximum platelet production of TxA2. A reduction in sTxB2 concentrations to below 10 ng/mL is uniformly associated with ≥95% inhibition of arachidonic acid-induced platelet aggregation. Therefore, sTxB2 levels of ≥10 ng/mL after aspirin treatment are usually taken as the threshold for a suboptimal aspirin effect [54]. A 2015 study by Baggen et al. showed that only determination of the sTxB2 concentration in clotted whole blood demonstrated the ability to measure activated platelets that generate thromboxane by activating COX-1. Therefore, the sTxB2 test is considered the most accurate and appropriate method for assessing the pharmacological effects of aspirin and is also the most stable and reproducible test for determining the response to aspirin. The 10 ng/mL threshold has often been chosen to define an adequate response to aspirin as sTxB2 concentrations below this level are associated with >98% inhibition of COX-1 activity in platelets in healthy subjects taking 100 mg aspirin daily [55].

### 2.7. The Importance of ASA in the Treatment of Cardiovascular Diseases and Stroke

The effectiveness and usefulness of ASA can be seen in the treatment of peripheral artery disease (PAD). Taking acetylsalicylic acid in combination with clopidogrel significantly reduces the risk of stroke, heart attack, and death from vascular causes in patients with PAD. A study by the Physicians’ Health Study found that low-dose ASA use reduced the risk of peripheral artery surgery by 54% compared with a placebo. It is worth emphasizing, however, that taking acetylsalicylic acid at higher doses, i.e., above 325 mg/day, often causes disorders of the gastrointestinal tract. In order to reduce the risk of atherosclerotic complications and vascular death, all people with peripheral arterial atherosclerosis should use ASA or another antiplatelet drug in low doses, unless there are contraindications [56].

From the neurological point of view, acetylsalicylic acid is important, and the legitimacy of its administration is not in doubt in the acute phase of stroke in patients for whom thrombolytic treatment cannot be initiated. The initial loading dose is 150–300 mg. After thrombolytic therapy, it is recommended to implement ASA after 24 h. However, there are also no specific decisions as to what dose of ASA is optimal for continued therapy for secondary prevention of stroke. The guidelines state that the recommended daily dose of ASA is 50–325 mg. Combining ASA (25 mg) with dipyridamole (200 mg) twice daily or clopidogrel 75 mg daily is also effective. In a group of patients with recent minor non-cardioembolic ischemic stroke or high-risk TIA, dual antiplatelet therapy (aspirin plus clopidogrel) should be started early and continued for 21 to 90 days, followed by single antiplatelet therapy [56]. Studies that examined ASA doses of 300 mg/day and 1200 mg/day did not show a better effect of the higher dose. A preventive measure recommended after an ischemic attack or acute stroke is taking ASA at a dose of 160–325 mg/day, and after a time, in the case of chronic protective therapy, it is recommended to use lower doses [57]. An extended-release formulation of aspirin developed to address some of these limitations recently came out. This may be an alternative to standard aspirin in the secondary prevention of cardiovascular disease because of the decreased bleeding risk of the therapy in conjunction with potent P2Y12 receptor blockers and/or oral anticoagulants [58].

### 2.8. Incomplete Response to ASA Consumption 

A lack of positive laboratory or clinical effects of ASA ingestion is termed ASA resistance. After taking ASA, a significant proportion of people react in a similar way in ex vivo measurements of thromboxane production or platelet aggregation. Nevertheless, 10–25% of people seem to have an incomplete response to ASA consumption [39]. Other researchers state, however, that they make up 5–45% of the population [59]. Multivariate analysis in the Taiwanese population revealed that acute stroke presentation was the only factor associated with aspirin non-responders, and that resistance was seen in nearly 50% of stroke patients [60]. The term “ASA resistance” does not have a strict medical definition, as it has not been accurately described or adequately standardized from a clinical point of view. Individuals defined as resistant to laboratory ASA showed a reduced inhibition of thromboxane levels as well as platelet aggregation or activation after taking ASA, as determined by laboratory or biochemical tests. It should be borne in mind that in the case of ASA, as with any other drug, one must take the dose of the drug, interference, and insufficient patient compliance into account. Other causes of the abnormal ASA response have also been described, including stress-induced inflammation and oxidation [40].

In a study by Lopez, it was shown that if a patient is not taking aspirin and the level of 11-dehydro-TXB2 is below 2500 pg/mg, no action is necessary. If the level of 11-dehydro-TXB2 is between 2500 and 10,000 pg/mg, then for the ASA response, administration of ASA should be considered and the main factors of cardiovascular diseases should be considered. However, when the level of 11-dehydro-TXB2 rises above 10,000 pg/mg, patients should be given ASA in order to assess the ASA response and to look for other risk factors of cardiovascular diseases. However, if the patient is receiving aspirin and the concentration of 11-dehydro-TXB2 is below 1500 pg/mg, no action is required because of the good ASA response, although monitoring of the risk of cardiovascular events is recommended. At concentrations above 1500 pg/mg, the patient shows an inadequate response to acetylsalicylic acid and factors such as patient compliance, adequate ASA intake, and additional antiplatelet therapy should be considered [40].

In summary, this algorithm is based on the fact that a continuous high baseline level of 11-dehydro-TXB2 in patients who do not take ASA may be a determinant of testing and observing the underlying risk factors for cardiovascular disease. However, the increased risk of atherosclerosis is only indicated by the presence of high levels of 11-dehydro-TXB2 after ASA. This indicates the need for comprehensive management, including antiatherosclerotic and antiplatelet therapy [61].

### 2.9. Other Methods of Blocking the TX Synthesis Pathway and Treating CVD

Other methods are also used to treat CVD, including stroke. Long-term use of non-selective NSAIDs (etodolac, nabumetone, ibuprofen, or naproxen) is associated with an increased risk of serious treatment-related cardiovascular events (acute myocardial infarction, angina, cerebrovascular attack, and/or transient ischemic attack) compared with long-term use of celecoxib. The mechanism of action of celecoxib is to inhibit the activity of cyclooxygenase, where the affinity for COX-2 is 375 times greater than the affinity for COX-1 [62].

The effect of terutroban, which is a selective antagonist of the thromboxane receptor, was tested. It blocks platelet aggregation and vasoconstriction induced by thromboxane; however, this drug has not been shown to have a better effect on the progression of carotid atherosclerosis. Sodium ozagrel, a thromboxane A2 synthase inhibitor, is one of the most studied drugs that may reduce the risk of neurological impairment and reduce the volume of brain damage [63]. Moreover, both dazoxiben and ozagrel show a possible cation-π interaction between the iron atom of the heme group of thromboxane synthase (TXAS) and the basic nitrogen atom of the imidazolyl group of these inhibitors [64].

**Figure 2:**
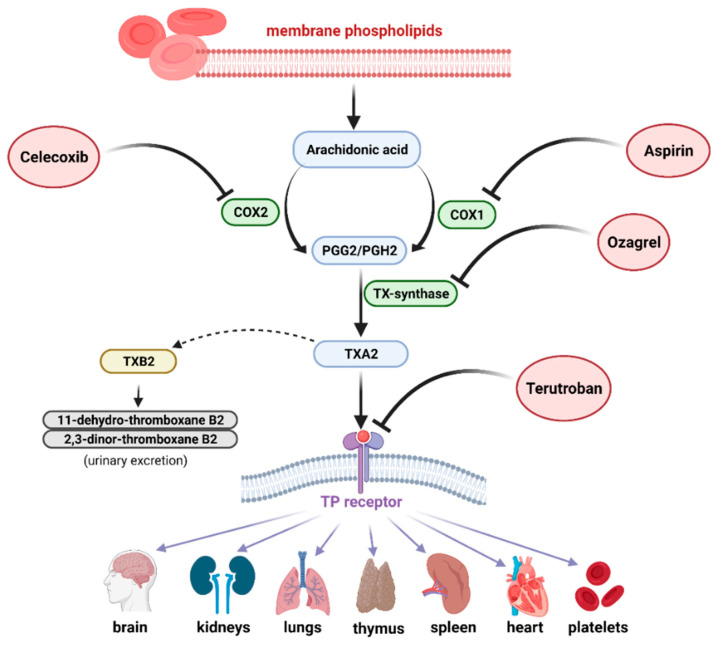
The scheme of action of thromboxane antagonists: aspirin is an inhibitor of cyclooxygenase 1 (COX-1) and celecoxib inhibits COX-2. Ozagrel blocks the conversion of PGH2 to TXA2 by inhibiting thromboxane synthase (TXS). Terutroban (TP antagonists) block the activation of the thromboxane receptor (TP receptor) (created with BioRender.com).

It has also been shown that thromboxane A2 production was inhibited by estrogen, and the effect was blocked by the G protein-coupled estrogen receptor (GPER)-selective antagonist of G36 [65]. A diagram of the action of drug inhibitors of the thromboxane synthesis pathway is presented in Figure 2.

## 3. Conclusions

The effectiveness of ASA in the prevention of ischemic stroke can be determined by determining its biologically active substance, which is thromboxane. The studies examined here showed that an initial high concentration of 11-dehydro-TXB2 may be a risk factor for ischemic stroke or ischemic heart disease. However, there is currently insufficient evidence to suggest that thromboxane can be used in hospital practice as a marker of ischemic stroke. The first drug in the treatment of stroke should be acetylsalicylic acid; however, its insufficient power in 25% or even 50% of the population should be taken into account. An alternative and/or additional therapy could be the use of a selective antagonist of the thromboxane receptor. Thromboxane A2 production is inhibited by estrogen; therefore, the risk of CVD after the menopause and among men is higher. Natural substances such as polyphenols, catechin, and cinnamic acid derivatives can be used in the prevention of CVD, but not in treatment.
